# Construction of complex memories via parallel distributed cortical-subcortical iterative integration

**DOI:** 10.1016/j.tins.2022.04.006

**Published:** 2022-05-19

**Authors:** Neil McNaughton, Seralynne D. Vann

**Affiliations:** 1 Department of Psychology and Brain Health Research Centre, University of Otago, POB56, Dunedin, New Zealand; 2 School of Psychology, Cardiff University, Park Place, Cardiff CF10 3AT, UK

**Keywords:** Memory, iterative processing, anterior thalamic nuclei, cerebellum, mammillary bodies, supramammillary nuclei, theta

## Abstract

The construction of complex engrams requires hippocampal-cortical interactions. These include both direct interactions and ones via often-overlooked subcortical loops. Here, we review the anatomical organization of a hierarchy of parallel ‘Papez’ loops through the hypothalamus that are homologous in mammals from rats to humans. These hypothalamic loops supplement direct hippocampal-cortical connections with iterative re-processing paced by theta rhythmicity. We couple existing anatomy and lesion data with theory to propose that recirculation in these loops progressively enhances desired connections, while reducing interference from competing external goals and internal associations. This increases the signal-to-noise ratio in the distributed engrams (neocortical and cerebellar) necessary for complex learning and memory. The hypothalamic nodes provide key motivational input for engram enhancement during consolidation.

## What is memory for?

Memory is phylogenetically old. Important aspects of human memory are ancient: they are present in simple invertebrate circuits [1]; well-developed in fish; and strongly homologous in birds [2]. Therefore, human cortical memory has emerged out of conserved [3] fundamental subcortical memory systems. While basic memory systems are present across species, memory capabilities have expanded with evolution, requiring a more energy-expensive brain. However, neural memory did not evolve to simply store data; its adaptive functions are linked to motivation. The bringing to mind of past experience, and of past goal-subgoal sequences [4], generates current goals and the means to achieve them. Yet, many assume that memory control is in ‘cold’ neocortex rather than in ‘hot’ limbic [5-8] cortex; and certainly not in ultra-hot ‘survival circuit’ [9-12] subcortex. We think these assumptions need to be revisited.

Here we argue that formation of even the most data-focused engrams^1^ in the cortex depends on a set of highly conserved nested cortical-subcortical-cortical closed loops that are essentially a set of parallel Papez circuits [14]. These loops support iterative processing – paced by inhibitory ‘theta’ rhythmicity – and are positioned to add motivational bias. Critically, wherever activity is blocked within these loops, the outcome is similar neural and behavioral dysfunction. The same Papez architecture can be seen across amniotes and, likely, monotremes and Theria [15], with perhaps a single equivalent loop even in fish [16-19]. However, there is also evolutionary progression. Relative to other mammals and primates, the most recent of the diencephalic Papez-like closed-loop components appears larger in humans alongside the relatively expanded telencephalon [20]. This Papez architecture is at least partially distinct from, and more unidirectional than, the many other interactive loops that use a “hierarchical system of brain oscillations” [21] to support more global processing including the “emergence of cognition from action” and alternative event predictions (see Fig. 1 in [22]).

## How and why do old brain areas control recent ones?

A key to goal-oriented engram processing lies in the motivation-biased base of the brain. The hypothalamus is a surprisingly important node in mnemonic loops [23]. At only 2% of brain volume in rodents (0.3% volume in humans) [24,25], it is nonetheless key to a vast range of functions. It controls not only low-level autonomic and homeostatic functions [26], but (often overlooked) high-level cognitive ones [27]. It is small and so, must exert wide-ranging diffuse control – not supply detailed computation. Importantly, hypothalamic nuclei can add *emotional bias* to the loop processing of engrams – held in areas such as the cortex – and so have a major adaptive impact on memory.

**Figure 1:**
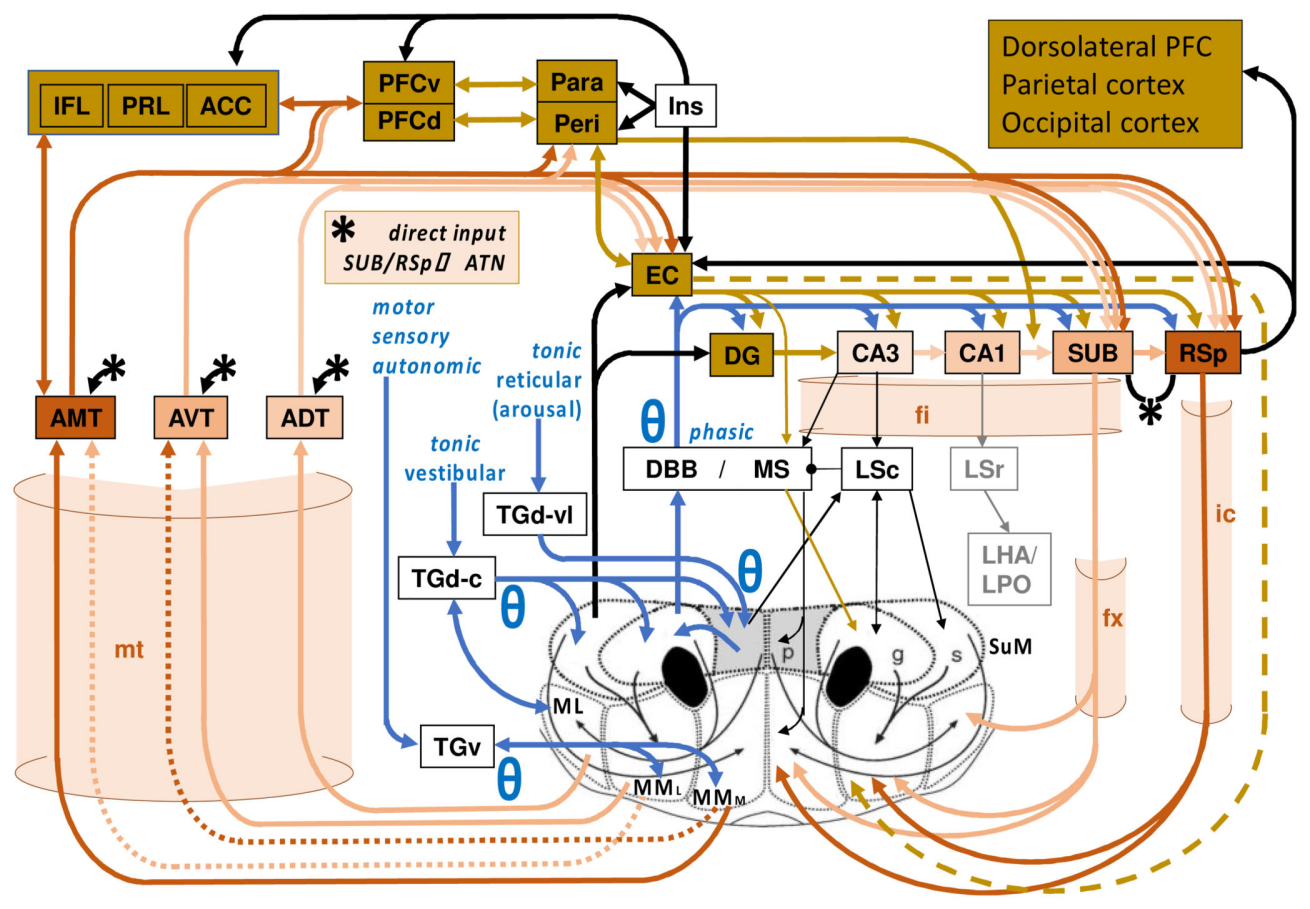
Overview of long and short loop connections from the hippocampus via mammillary area to the frontal cortex and back present in mammals ranging from rodents to primates [28,51,71,123-128]. The mammillary bodies (MB) and supramammillary area (SuM) have aligned medial, mediolateral, and lateral parts. MB targets prefrontal and anterior cingulate cortex, which target the hippocampal formation, completing the Papez circuit. Tonic arousing reticular input to medial (p = parvicellular [28]) SuM is converted to phasic theta rhythmicity (**θ**), passed to mediolateral (g = grandicellular) SuM, then diagonal band of Broca (DBB)/medial septum (MS) complex then hippocampal formation. Lateral (s = shell) SuM project to entorhinal cortex (EC). The fimbria (fi), fornix (fx), and internal capsule (ic) return hippocampal formation output to SuM/MB in onion-like, nested loops. EC, dentate gyrus (DG), CA3, CA1, subiculum (SUB), and retrosplenial cortex (RSp) connect unidirectionally. Successive loops are similar, but outside loops have greater delays and more highly processed information. There is a similar ‘onion’ with mammillothalamic tract (mt) output from MB and output from AMT/AVT/ADT to infralimbic (IFL), prelimbic (PRL) and anterior cingulate (ACC) cortex. Dorsal and ventral prefrontal (PRFd, PRFv) then perirhinal (Peri) and parahippocampal (Para) cortex complete the Papez circuit in EC. We have not included, e.g., the AMT-CA1 connection [129], to keep the fundamental architecture of the loop circuits clear. Abbreviations: ADT, AMT, AVT = anterior thalamus, dorsal, medial, ventral, respectively. ML, MM_L_, MM_M_ = mamillary nucleus, lateral, medial pars lateralis, medial pars medialis, respectively.

Two adjacent posterior hypothalamic areas are particularly linked to cognition: the mammillary bodies (MB) and supramammillary area (SuM). Each was initially seen as homogenous – but both have three distinct, matching, parts [28]. The six parts differ in detailed anatomical connectivity and, at first sight, functions. Discrete lesions, targeting of specific connections, and genetic models (particularly in SuM) have separated contributions from subregions as well as structures [29-34]. For example, the medial MB and lateral SuM both contribute to hippocampal activity during REM sleep [35,36]. Lateral and medial MB lesions both impair performance on spatial memory tasks, but the pattern of impairments is different [30,37]. Lateral SuM is thought to have a greater role in spatial learning and memory [33] with medial SuM being more biased towards inhibitory learning [38]. Nonetheless, all six parts have similar roles in the integration of input from ascending activating (‘arousal’) systems and in the rhythmic pacing of processing in, e.g., the Papez circuit [14]. As noted above, SuM and MB are physically adjacent, and we suggest that they are also computationally similar – but as key nodes in distinct loops that have more engaged subcortex and cortex, respectively (see Figure 1). They provide subcortical motivational input into parallel circuits that support hippocampo-cortical long-loop interactions.

## Memory *and* Emotion?

Papez [14] initially put forward his eponymous hypothalamically-mediated hippocampus-cortex-hippocampus circuit as the basis of emotion [23]. However, to later researchers, the role of the hippocampus in amnesia (particularly obvious in Henry Mollaison [39]) made the circuit seem more relevant to memory. However, memory versus emotion is a false dichotomy, given that goals require both, and hippocampal damage alters emotion [40-42]. Indeed, the hippocampus is among the main structures controlling the level of stress (and other) hormones [43-45]. Current descriptions of memory, and its processes, need to better reflect interdependence with emotion.

Lesions at any point within the Papez circuit (or its sub-loops) can impair memory. While the severity and specificity of memory impairment can vary according to site of pathology, a similar pattern of impairments can be seen throughout the system. Importantly, in both humans and rodents, many aspects of memory remain intact, e.g., in simple item discrimination tasks and procedural tasks [46,47]. Rather than affecting a particular type of memory [48], impairment usually requires that any type of paradigm have sources of interference – as when cues are combined into spatial features, complex objects, or temporal contingencies [e.g., 49,50]. But Papez circuit structures are also all implicated in stress, anxiety, and emotion – all can produce anxiolytic effects in standard tests of anxiety including approach-avoidance conflicts [51]. (Consistent with this, Henry Mollaison appeared to be unusually lacking in anxiety; J. Ogden, pers. comm.) Benzodiazepine anxiolytics can produce amnesia; the high density of benzodiazepine receptors within both the MB and SuM could contribute to these amnestic effects [52,53].

The MB and SuM receive external representations via inputs descending from limbic, temporal, and prefrontal cortices. But their key role is integrating these representations with ascending somatic inputs. For instance, both regions have cells strongly responsive to running speed and they moderate hippocampal speed-cell function [54-56]. They are also able, via inputs from the dorsal tegmental nucleus, to provide wider hippocampo-cortical circuits with vestibular input that is crucial for spatial memory (including hippocampal theta rhythm and ‘place fields’ [57,58]).

Lower-level input to memory circuits is not functionally trivial. Simple *peripheral vestibular receptor* damage disrupts emotion and memory: it is associated with hippocampal atrophy and may be a risk factor for dementia [59]. Other low-level inputs (including from the ventral tegmental nucleus) provide sensory, motor, autonomic, and arousal-related information and control the frequency of hippocampal theta pattern activity. The theta pattern, *per se*, is important for neural plasticity [60] and spatial learning [61]. But, its disruption does not change the basic organisation of place fields [62], unlike disruption of head direction pathways in the antero- or lateral-dorsal thalamus [63,64]. The theta system also responds strongly during threat-induced freezing [65].

In the context of goal processing, neocortex (particularly the interaction of anterior and posterior neocortex) can maintain representations of the external world but, we argue, would need the ascending inputs from the base of the Papez circuit to add key ‘contextual / emotional / internal’ information. In particular, if *episodic* memory (and mental time travel) depends on the cell assemblies originally postulated by Hebb [66], spatial and temporal direction could be added by SuM/MB theta-rhythmic control during circuit processing. The need for internal direction inputs for event processing would explain the importance of simple vestibular input for ‘memory’. The ascending inputs to SuM would therefore inject position/emotion information into the base of the Papez circuit, while also having more direct connections to higher levels including the hippocampus.

## Why is memory controlled by iterative loops?

The original circuit envisioned by Papez [14], while capturing the essence of a key *unidirectional* loop around the various regions, does not give an accurate representation of the multiple parallel Papez-architecture closed loops that it encompasses (Box 1; Figure 1) and that all have their lowest nodes in SuM/MB. The length and number of these closed loops likely explain the fact that the range of frequencies of the (usually synchronous) theta rhythmicity covers the round trip time from cortex to subcortex and back for these circuits [67,68]. One of the shortest is SuM➔hippocampus➔MB➔SuM; where iteration has been clearly demonstrated and directional control of the rhythmicity shown to vary with its acceleration and deceleration [see 69]. Multiple loops also fit with suggestions that the hippocampus uses “big loop” iteration^2^ for not only episodic memory but also “integration of information across experiences” [70, p. 1342] and likely other forms of inferential processing.

On first glance, the circuits give an impression of redundancy. For each indirect two- or three-synapse connection in a single pathway, there is usually a direct single synapse connection – with the direct and indirect paths often starting as collaterals of the originating neurons [71].

Why would this be, computationally? These multi-level connections provide a means for multi-level processing. Each pathway is one part of a hierarchical onion-like layering, where a simple direct first pass through subcortical ‘survival circuits’ [9] (evolutionarily early, conserved, and more likely linked to encoding and engram formation [72-74]) is followed by progressively more complex indirect cortical processing (evolutionarily late, expanding, and likely linked to recall, consolidation, reconsolidation, and perhaps more recently even imagination [75-77]). In evolutionary terms this subcortical/cortical hierarchy allows integration of fast but reflexive with slow but sophisticated processing [78] – achieving, phylogenetically, the most efficient processing across a range of task urgencies.

We think there are two, linked, issues here. The prime issue for an evolved system is a form of Cocktail Party Problem. How to separate signal (situation-specific, not necessarily loud) from noise (which may be loud and also situation-related) via motivational bias. You must detect what you most need not what is most salient. This is analogous to the classic figure-background problem, most easily solved in perceptual systems [79,80], where within-circuit iteration is computationally advantageous [79]; but with added active motivational filtering. Second, is the issue of recall. How is just one item retrieved against a background of similar competing remembered items? For both issues there is a need to prevent percept-level interference and catastrophic forgetting [48].

**Figure 2:**
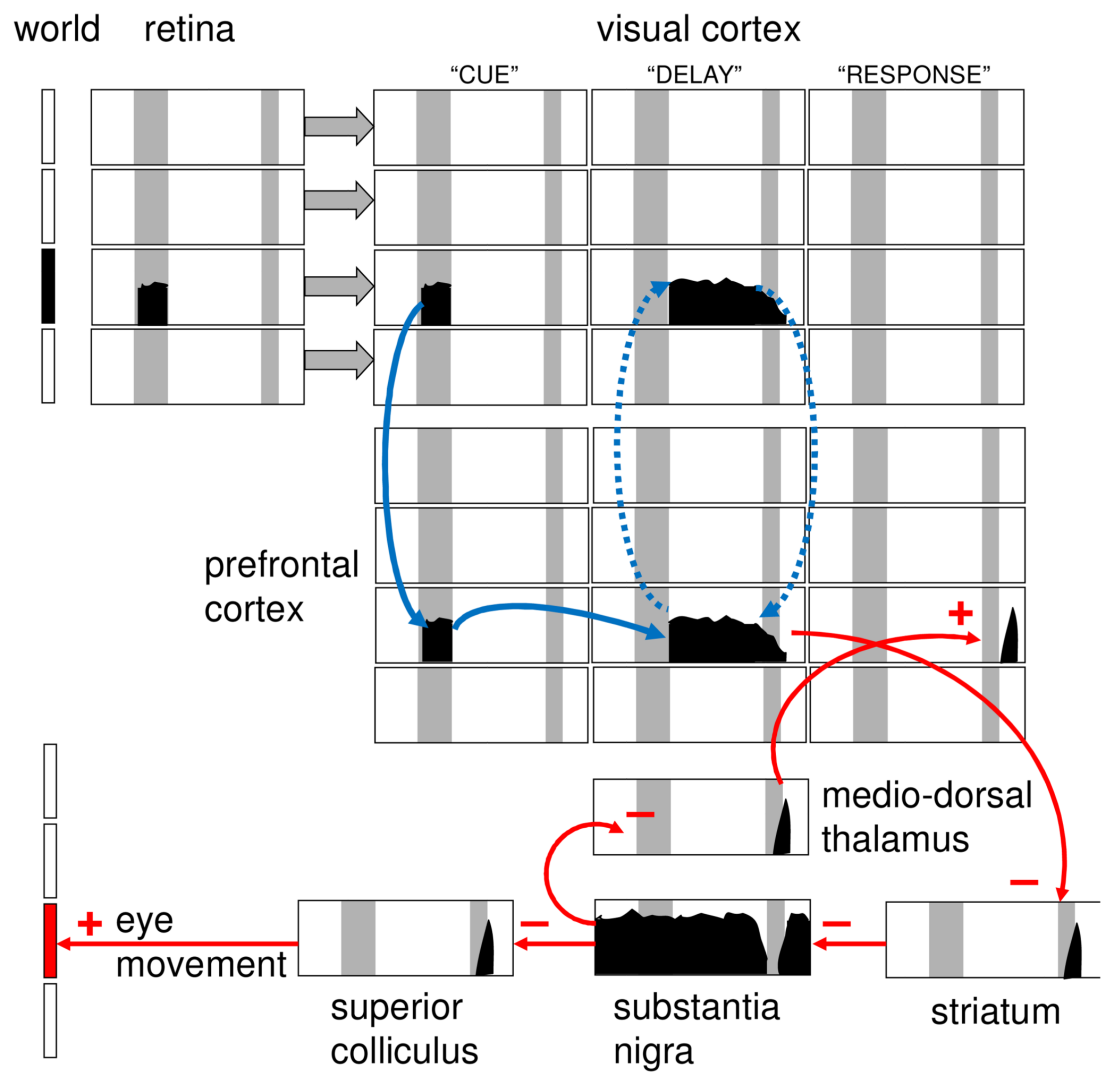
Perception and action are intertwined in a cycle in mammals. They share their neural circuitry [130,131]. The will to act must start with a goal, which is usually marked by an external percept. The percept, itself, may be fleeting, but then prefrontal cortex uses iterative loops [132] to hold information in posterior cortex in the form of active memory [133]. The figure illustrates these general principles with a simple example based on a delayed response working-memory task in monkeys [132]. A target position is briefly indicated on a screen and registered by the retina (top left) which passes information to visual cortex, which in turn activates prefrontal cortex. During a delay interval, activity from prefrontal cortex refreshes visual cortex, keeping the stimulus location in active memory. When the end of the delay interval is signalled, this location is read out to circuits controlling eye movement and the monkey then looks at the position where the target was before the delay. Note that, unlike trace conditioning tasks, delay tasks do not depend on hippocampal circuitry. Figure adapted from [51] with permission.

We suggest that each of the known parallel loops operates to separate key percepts and engrams from interfering associations and alternatives in much the same iterative way as a figure is separated from its ground [79] but by motivational filtering (at the SuM/MB nodes). Current *active* memory – one of the earliest stages of processing – is known to hold information without modification by simple iteration in frontal-posterior loops (Figure 2). In contrast, we suggest that Papez-architecture loops through MB/SuM *modify* engrams via iterative reprocessing. This iterative reprocessing *progressively* enhances active circulation of target stimulus components, while suppressing active circulation of interfering stimulus components, through application of a *motivational* filter. This would, in the first instance, reduce confusing competing associations from non-target external stimuli.

An initial engram would usually be a simple cell assembly [66]. Both consolidation and repeated experience will then add additional components to this original engram and generate distributed engram ensembles [13]. The MBs provide theta pattern input that guides plastic engram formation, both in the hippocampus and the cortex [35,81,82]. Functionally, damage to the MBs, and other regions within the basic Papez circuit, is associated with relative impairments in recollective memory while ability to discriminate whether simple items have been previously experienced (i.e., familiarity) is left intact [e.g., 49]. These dissociations are often couched within dual-processing models: two functions that are distinct and dissociable. However, we suggest that this pattern of impairments instead reflects differences in the current configuration of the cell assembly coding the engram, not on distinct processes. That is, for complex episodic information, activation of the single *correct* associative representation is necessary for recall. In contrast, the activation by perceptual input of a more impoverished engram would be sufficient to detect a previously experienced item. As such, a nascent partial engram would be detectable as familiar even when the final full engram is not yet sufficiently developed to be recalled against the background of interference. Although it is implicit in the ideas of consolidation and reconsolidation, we think that engrams are rarely seen as both unitary and dynamic to this extent. But iteration [79] (particularly via replay [83-86]) combined with more basic interactions between Hebbian learning and single-cell homeostasis [87] can solve the problem of retaining viable (albeit labile) memories in a dynamic world.

Anatomically, there are multiple loops. Each of these loops operates at its own hierarchical neural level (note the increase in loop nodes as one goes from ADT to AVT to AMT in Figure 1). The brain uses parallel reflexive versus slow complex processing in many systems [78] to balance urgency against clarity. Papez looping can start in a shorter loop, and then be accompanied by processing in progressively longer loops (Figure 1). With sufficient time (via consolidation or repeated experience) this would selectively enrich the engram by allowing more distributed cell assemblies. Such enrichment could involve extension of the time across which traces can be maintained [88], or later, extension of the components of the engram with more experience, or consolidation, or reconsolidation [86], during wake or sleep [35]. Simple *repetition* of information in each loop as in active memory would enhance the strength of connections of a cell assembly through conventional Hebbian processes [89,90] for both noise and signal. By contrast, active iterative *reprocessing* (combining general enhancement with selective suppression of motivationally unwanted connections [91,92]) should allow complex (e.g., temporally and episodically related) engrams to become stabilized in the cortex. So, improvement in signal-to-noise ratio would not just be through reduction in noise but also through creation of a richer signal through expansion of the engram by progressively more sophisticated loops. Consistent with this, lesions of the mammillothalamic tract in rats reduce expression of plasticity markers in the retrosplenial cortex [81,93], reduce neurogenesis and spine density in the hippocampus, and reduce long-term grey-matter changes observed in both hippocampus and cortex following spatial training [35].

## What triggers iterative processing?

Cortex-subcortex loop-related integration is an adjunct to the distinct cortex-cortex looping that maintains active memory. Active memory is refreshed by loops in which frontal and posterior cortex can be seen as simple relays. (The refreshment cost is born by the fact that information needs to be temporarily maintained for adaptive function.) But cortical-subcortical loops go beyond, and should not be confused with, the simplicity of loops underlying active memory. The temptation is to see prefrontal cortex (highly expanded in humans relative to other species) as a be-all and end-all. This is an (anthropocentric) error.

“To give to our prefrontal cortex the role of the autonomous origin of all our decisions and actions leads inevitably to an infinite regress that should be avoided (“What agency controls the prefrontal cortex? What other agency controls that one?”…and so on ad infinitum). The only reasonable solution to the quandary is to place the prefrontal cortex in the perception-action cycle, where the action can originate anywhere, including the cerebral cortex, prefrontal or other.” [94, p. 7]

The perception-action cycle depends on the interaction of anterior with posterior cortex (for an example, see Figure 2). But a goal requires not only situation (whether a local object or a more complex context) but also motivation (a neutral object will not be a goal). To some extent motivation will be supplied by limbic cortex via its circuits with the prefrontal cortex. However, output from these areas, via the Papez circuit, to the hippocampal formation then receives hippocampal and hypothalamic processing before returning in a modified form to the prefrontal cortex via the thalamus [95]. Functional hippocampal output requires that it receives theta pattern input from the medial septum, dependent on arousal-related reticular and cerebellar input via areas such as SuM [61]. The passage of the resultant hippocampal output through the MB appears to depend on similar arousal-related inputs from the dorsal and ventral tegmental nuclei [96-98]. Thus SuM/MB would provide arousal and interoceptive information to boost and bias iterative processing, while prefrontal cortex would contain the cell assemblies that the process enhances; with increased signal/noise ratio with each iteration. The result would be truly iterative processing (unlike the simple echo of active memory) with all nodes in a loop able to adjust their output.

## Does iteration affect cerebellum as well as neocortex?

**Figure 3:**
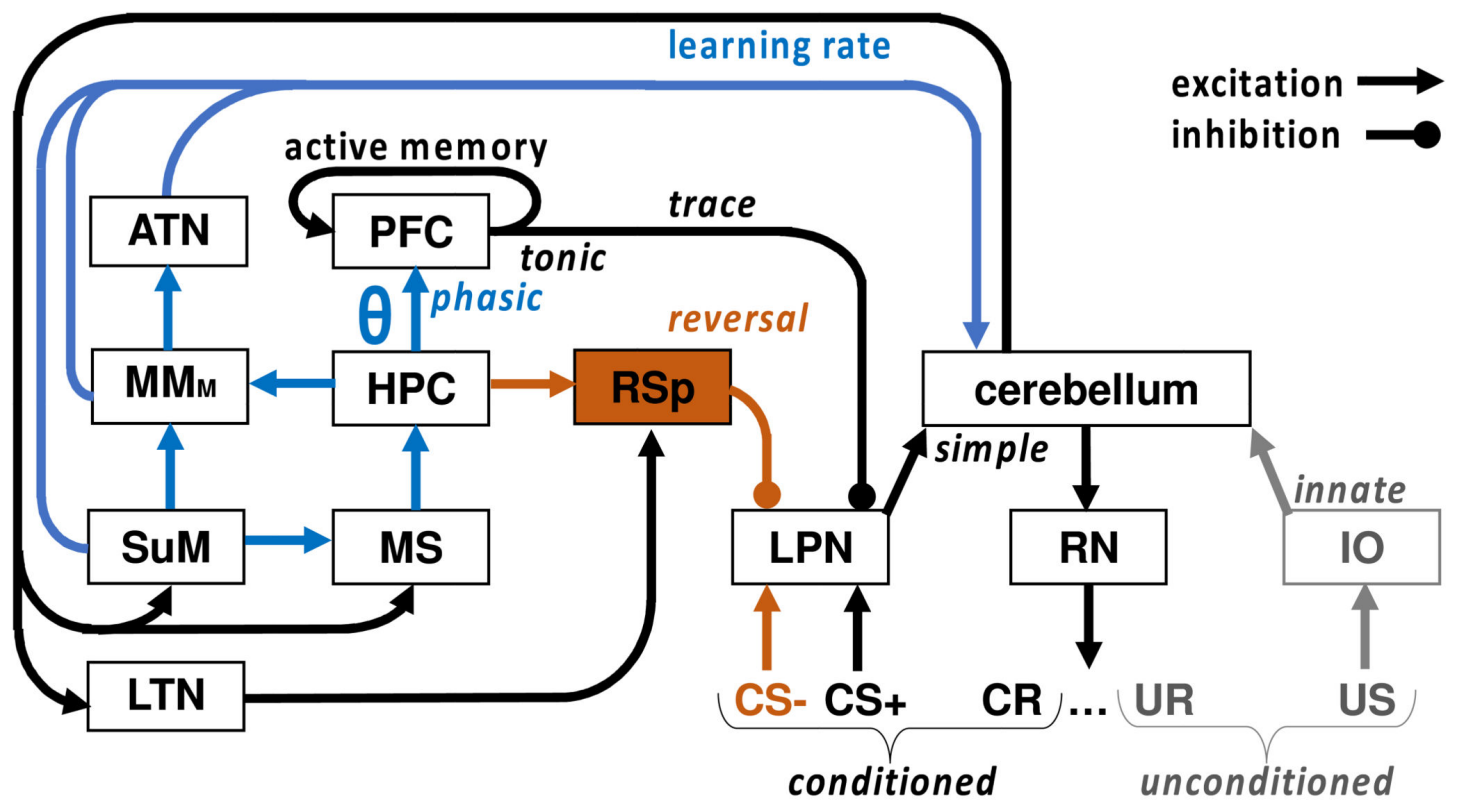
The role of the hippocampus (HPC) in eyeblink conditioning in mammals, based on [134-138]. HPC cells show firing patterns – triggered by the conditional (CS) but not unconditional (US) stimulus – that arise in training, progress during conditioning, and often model the conditioned eyeblink response (CR). Note that this combination of stimulus control with response-related firing implies that the hippocampal circuit is processing complex goal information rather than simple stimuli or actions. Hippocampal lesions do not affect simple, or delayed, or discriminative (CS+/CS-) conditioning. However hippocampal lesions affect both trace conditioning and discrimination reversal learning. Trace conditioning is mediated via output from delay-line activity from prefrontal cortex to lateral pontine nuclei (LPN) that inhibits activation of the eyeblink by the CS+ (in this case there is no CS-). Reversal is mediated via output from the retrosplenial cortex (RSp) that inhibits activation of the eyeblink by the CS- (which was the CS+ until reversal was started). Hippocampal theta-related output from HPC via the supramammillary nucleus, medial mammillary nucleus (MMM), and anterior thalamus (ATN) via pontine nuclei [139], impacts rate of learning [138]. Note that, in humans, “comparable delay and trace activation was measured in the cerebellum, whereas greater hippocampal activity was detected during trace compared with delay conditioning” [140] and there is good evidence for involvement of such cerebellar circuits in working memory generally [99].

The hippocampus is connected by closed loops to not only neocortical areas but also to the cerebellum; as are the cerebellum and neocortex [99,100]. The cerebellum’s contribution to memory was traditionally considered limited to motor learning. In this context, eye blink conditioning provides a well-studied example of both the cerebellar role in simple conditioning and its interaction with limbic structures in, e.g., trace conditioning (Figure 3). However, there is increasing evidence that cerebellum has a more widespread, cognitive and emotional, role.

One proposal is that the cerebellum has a particular role in goal/reward learning, especially in novel situations. Cerebellum supports this function using trial-by-trial error-correction, similar to its contribution to motor learning [101] – here its involvement is akin to the interference reduction seen with more cognitive engrams. Situation (coupled with motivation) is a key element of goals. It is most easily understood by experimenters when a situation reflects a place. The hippocampus, of course, has place (we would say goal) fields and the cerebellum contributes to the association of hippocampal place fields with objects by updating the place fields when the objects are re-located [102]. The synchronization of cerebello-hippocampal interactions is also necessary for appropriate spatial processing [103]. Overlapping similarities between MBs/SuM and cerebellum include contributions to hippocampal processes for goal/spatial learning [102], involvement of theta [103], and a bias for processing temporal information [50,104,105]. The unexpected hippocampal role in eating [40-42] is also echoed by the cerebellum [106]. The direct connections between MBs/SuM and the cerebellum form additional, remarkably similar, loops that provide further inputs required for the development of representations to support long-term hippocampal-dependent learning when, as with trace eyeblink conditioning, this involves the cerebellum.

More general computational features of interest in the cerebellum include [107, for review]: 1) its different implementations of learning at different timescales; 2) its greater involvement during the first hours after learning; 3) extensive recurrent connections allowing iteration; and 4) apparent similarity of computations across areas, with differences in functional output depending on the specific other brain areas providing input and receiving output. All of these features are reminiscent of extended hippocampal circuits.

“A key difference between the cerebellum and other brain areas is the extraordinary amount of neural hardware devoted to input preprocessing in the cerebellum, which is roughly equal to the number of neurons in the rest of the brain combined. Yet the computational functions that have been attributed to the cerebellar preprocessing stage are similar to those that have been described for other brain areas — decorrelation, pattern separation, and the generation of temporal basis sets.” [107, p. 244]

Importantly, while already clearly present in species like sharks that have no neocortex, the cerebellum has steadily developed and expanded in phylogeny. In primates and humans, in particular, its expansion has been *greater* than that of the neocortex. Compared to baboons, human cerebellum is 15% larger than would be expected from the expansion of neocortex; and this cerebellar expansion is likely to have contributed to human cognitive evolution through increased technical intelligence, advanced technological capacities, and preadaptation for language [108]. Recent studies have also indicated a role for the cerebellum in both the perception and the action components of active memory [109]. As such, the perception-action cycle appears to be supported by distributed networks from neocortex to cerebellum, including Papez-architecture circuits that contribute both directly and indirectly [99,100].

## Concluding remarks and future perspectives

Construction of complex memory engrams engages widespread cortical-subcortical networks. Even simple engrams may require extended processing (consolidation, reconsolidation) under conditions of extinction or reversal. Here, we have focused on the contribution of two adjacent hypothalamic areas, MB and SuM, to their Papez-architecture circuits.

We have reviewed data that demonstrate:

1. The presence of closed loops, which provides the capacity for iterative processing (Figure 1).
2. Iterative looping in, for example, the SuM➔hippocampus➔MB➔SuM circuit with evidence for driving of the circuit from SuM➔hippocampus during theta frequency acceleration and from Hippocampus➔MB➔SuM during deceleration [see 69].
3. The importance of the Papez-architecture circuits for interference reduction [e.g., 49,50].
4. The contribution of the Papez-architecture circuits to the formation of mnemonic representations in hippocampus and cortex [e.g., 35,110].
5. The integration of motivational and situational information into the circuits at SuM/MB (see Box 1).

We have combined these known features of the system to suggest that the Papez-architecture circuits *use* their known capacity for iteration to progressively adjust signal and noise [79,80] coded by cell assemblies and *so* both enhance engrams and reduce interference. Both MB and SuM have a driving role in the combining of representations of both internal and external information that is needed to identify and prioritise engrams. Their component nuclei are positioned so as to allow iteration within, and among, parallel distributed loops. Iteration provides an ideal mechanism for integrating local and long-range inputs and so constructing and integrating elements of complex (e.g., episodic) engrams; while also limiting the effects of external (e.g., competing objects) and internal (competing associative retrieval) interference.

While the original Papez circuit has been associated with memory processing for over 80 years [see 111], its precise role has been unclear. Many have seen it as a relay circuit, passively transferring information; but this ignores the massive energy cost of axons and nuclei. And to what ends would it simply relay information? The hippocampus and cortex already have direct connections, what use is an additional loop? And why are there, in fact, multiple, nested, loops – including links to cerebellum that are similar to those to prefrontal cortex (with cerebellum and cortex also highly interconnected)?

We argue that the extended parallel distributed system of Papez-architecture loops has two adaptive functions. First, it enables the integration of internal cues - emotional and positional - into hippocampo-cortical-dependent engrams. Second, the iteration of information around the circuit allows representations to be fine-tuned and enhanced in terms of detail, while also increasing the signal-to-noise ratio via a process analogous to figure-ground separation [79,80]. Iterative reprocessing helps to construct memory representations that have sufficient contextual information to reduce interference across similar overlapping experiences. But beyond that, it also provides additional gateways to influence and incorporate wider networks for learning including the cerebellum. This adds further spatial and temporal processing with, again, its local iterative looping enabling the formation of distinct, separable, representations.

Seeing memory networks as extended subcortically beyond primary hippocampal-cortical interaction is essential if we are to properly model the dynamic widespread neural activity of memory construction and consolidation (see Outstanding Questions). This extension poses new experimental challenges. Individual brain structures are just small windows into the processing and storing of information. Detailed analysis of network level activity is needed to understand the dynamic interactions across memory circuits that vary with time and task demand. The greatest insights will be obtained when complex circuit analysis and sophisticated behavioral paradigms are combined. However, at present, advances in behavioral analyses are not developing at the same speed as the tools needed for circuit analyses. It is also important to make use of tasks that can tap into cross-species processes, while capitalizing on the natural behavior of individual species.

One key future methodological challenge is that the subcortical structures of interest are small and deeply located. This makes it hard to identify neural activity originating from them in humans. Non-invasive recording of electrical activity (such as with EEG) does not pick up from deep sources; while techniques such as fMRI are limited in their spatial resolution, particularly for small deep structures. Invasive recordings (in patients with implanted electrodes for neurological treatment) have been made of some structures, for example, of the MBs and anterior thalamic nuclei [112,113]. These have identified cross-species similarities in oscillatory mechanisms, but there are few studies to date, and these only involve individuals with underlying pathology. Future improvements in human imaging should address some of these outstanding issues; and combining techniques such as fMRI and EEG could also be advantageous. Testing the hypotheses derived from animal work in humans, coupled with detailed analysis of circuits and task phases, should elucidate the processing implied by Figures 1-3. Critically, we think that analyses should assess the role of the multiple parallel networks that we know exist in some form across a wide range of species. As with task selection, a key to future progress will be the use of appropriate comparative neural techniques [114] that allow for species-specific (often cortical) expression while assessing species-general (often subcortical) processes.

The extended Papez circuits, including the MBs and/or the anterior thalamic nuclei have been implicated in several neurological disorders that are associated with memory impairments, e.g., Korsakoff syndrome, Alzheimer’s disease, Down syndrome, and hypoxic-ischaemic encephalopathy [115,116]. However, there is also increasing evidence for a role for the medial diencephalon in psychiatric and neurodevelopmental disorders [117-119], where memory impairments are present but also emotional dysregulation. Given the role we attribute to the supramammillary and mammillary areas in Papez circuit processing we think it is time for a closer look into overlaps between memory and emotion across mnemonic, neurodevelopmental, and psychiatric disorders.

## Box 1 Connections of SuM and MB – two hierarchies of iterative loops

Cortex-subcortex connections are complicated, involving sets of hierarchically organised parallel closed loops. The higher functional levels of the hippocampus proper (area CA1 and the subiculum/SUB) send direct input only to evolutionarily old parts of the frontal lobe (i.e., prefrontal allocortex). In contrast, the Papez circuit allows CA3 to control more evolutionarily recent frontal isocortex [14] via the MB ➔ anterior thalamic nuclei (ATN)^3^ ➔ frontal cortex path. This ATN projection also allows the MB wide influence on temporal lobe memory systems [121] including the entorhinal cortex, retrosplenial cortex (RSp), and SUB (completing the Papez circuit). The MB are much more than a ‘relay’ – integrating hippocampal outflow with independent mnemonic inputs from Gudden’s tegmental nuclei in the midbrain.

The nuclei of the MB are connected by an onion-like series of nested loops to parallel levels of the hippocampal formation (Figure 1). “The different levels are unlikely to exist simply so as to pass unaltered information on to one another; each must allow some particular transformation or integration of its inputs. We propose that the hippocampus is a system of logical gates which allows different types of information to progress to different points of the circuit and hence to produce (or in many cases not produce) outputs from different levels of the system subject to different conditions” [51, p221]. Importantly, the intra-hippocampal connections are all in the same single direction. So, the hippocampal formation is rectifying (forcing information to pass in only one direction in the loops), as are the connections from SuM to MB.

Important features of the hippocampal ‘onion’ are: a) *initial*, partially-processed, output is from area CA3, ‘relayed’ by the lateral septum, to SuM; while, b) the main *final* highly-processed output is from SUB/RSp direct to MB. The similarity of the parallel loops suggests similar computational function, but the specific connections suggest that SuM mediates reflexive [78] and SUB/RSp mediate sophisticated processing. That is, SuM controls subcortical processing, using partially-processed (CA3) information and with no neocortical efference copy. Fully processed information from SUB/RSp goes directly to the higher (goal processing) areas of prefrontal cortex and anterior cingulate; with an efference copy (often via collaterals [71] and with similar cell-firing fields [122]) going directly to MB as part of “slow and sophisticated” processing.

The Papez circuit is also onion-like. Lateral, medio-lateral, and medial areas of MB primarily project to anterodorsal, anteroventral, and anteromedial thalamus, respectively. Prefrontal cortex and perirhinal/parahippocampal cortex complete the circuit in entorhinal cortex. All three thalamic areas make return projections to entorhinal cortex, SUB, and RSp.

## Outstanding Questions

There are many hippocampus⬄cortex and hippocampus⬄cerebellum loops, with widely varying architectures, and important mammillary connections independent of hippocampus. How do the architecturally different loops through, e.g., nucleus reuniens, interact with the anterior thalamic Papez-architecture circuits described in the current article? How do the circuits through the neocortex compare to those through the cerebellum?

Other types of circuit exist for other purposes. Papez-architecture circuits *are not required* for learning and memory in terms of simple stimuli and simple motor responses (e.g., eyeblink or freezing) but become involved when interference from pre-emptive (trace conditioning) or competing (water maze, conditioning to ‘context’) responses must be suppressed. What qualitatively and/or quantitatively determines the threshold for requiring Papez-architecture processing? Is this linked to the need for inhibition? Does this vary across individuals?

Disruption of MB/SuM disrupts a number of electrophysiological signatures across hippocampus and cortex. We know that behavioural impairments can be ameliorated with artificial stimulation that reverses this disruption – but how general is this? Would theta-frequency stimulation be sufficient to reverse all impairments due to Papez-circuit dysfunction or would the stimulation need to be more nuanced, e.g., taking into account gamma rhythmicity and theta-gamma coupling?

Memory construction happens across different time frames: during the event, immediately following the event and during subsequent periods of sleep. MB/SuM circuits have been implicated in all stages. Is essentially the same iterative integrative computation carried out during these different stages or does it vary according to state of the constructed engram?

## Footnotes

1 We use the word engram to refer to any one of the distributed Hebbian cell assemblies that are thought to be basic units of memory [13].

2 The circuits can be referred to as recurrent but, computationally, ‘recursion’ is a different process than iteration.

3 Loops through, e.g. nucleus reuniens are also important [120] but beyond the scope of this article
